# Fatty Liver Index and Skeletal Muscle Density

**DOI:** 10.1007/s00223-021-00939-9

**Published:** 2022-01-13

**Authors:** Julie A. Pasco, Sophia X. Sui, Emma C. West, Kara B. Anderson, Pamela Rufus-Membere, Monica C. Tembo, Natalie K. Hyde, Lana J. Williams, Zoe S. J. Liu, Mark A. Kotowicz

**Affiliations:** 1 Deakin University, IMPACT – Institute for Mental and Physical Health and Clinical Translation, Geelong, VIC Australia; 2 Department of Medicine – Western Health, The University of Melbourne, St Albans, VIC Australia; 3 Barwon Health, Geelong, VIC Australia; 4 Department of Epidemiology and Preventive Medicine, Monash University, Melbourne, VIC Australia

**Keywords:** NAFLD, Skeletal muscle fat infiltration, Muscle density, Myosteatosis, Hepatic steatosis, Sarcopenia

## Abstract

Accumulation of fat in the liver and skeletal muscle is associated with obesity and poor health outcomes. Liver steatosis is a characteristic of non-alcoholic fatty liver disease (NAFLD) and myosteatosis, of poor muscle quality in sarcopenia. In this study of 403 men (33–96 years), we investigated associations between the fatty liver index (FLI) and muscle density, as markers of fat accumulation in these organs. We also investigated associations between the FLI and parameters of sarcopenia, including DXA-derived appendicular lean mass (ALM) and handgrip strength by dynamometry. Muscle density was measured using pQCT at the radius and tibia. FLI was calculated from BMI, waist circumference, and levels of triglycerides and gamma-glutamyltransferase. There was a pattern of decreasing muscle density across increasing quartiles of FLI. After adjusting for age and lifestyle, mean radial muscle density in Q4 was 2.1% lower than Q1 (*p* < 0.001) and mean tibial muscle density was 1.8% lower in Q3 and 3.0% lower in Q4, compared to Q1 (*p* = 0.022 and < 0.001, respectively). After adjusting for age and sedentary lifestyle, participants in the highest FLI quartile were sixfold more likely to have sarcopenia. In conclusion, our results suggest that fat accumulation in the liver co-exists with fat infiltration into skeletal muscle.

## Introduction

Deposition of ectopic fat in the liver (liver steatosis) and infiltration of fat into skeletal muscle (myosteatosis) are both associated with impaired physiological function [1] and poor health outcomes [2]. Liver steatosis is a characteristic of non-alcoholic fatty liver disease (NAFLD) [2], and myosteatosis is a characteristic of poor muscle quality in sarcopenia [3]. The fatty liver index (FLI) was developed as a clinical risk assessment tool for identifying NAFLD. As intramuscular fat content increases, muscle density decreases, as the radiological density of fat is lower than muscle [4–6]. Sequelae of fat infiltration into skeletal muscle include muscle atrophy, reduced muscle mass and muscle dysfunction [3, 5, 7, 8], and higher mortality [9]. This is due, at least in part, to the adipose tissue in muscle producing and releasing adipokines and inflammatory factors that act locally and systemically [10]. These can be released into the circulation, where they contribute to low grade inflammation. Locally, the accumulation of intramuscular lipid disrupts cellular energy homeostasis and leads to reduced protein synthesis, calcium imbalance and loss of contractile function, strength and exercise capacity [10].

Whilst there is evidence to support skeletal muscle-liver crosstalk [11] and data to suggest an association between skeletal muscle volume and NAFLD [12–17], data looking at the association between liver fat and skeletal muscle fat are limited [18, 19]. If such an association exists, it might suggest that there are common mechanisms underpinning sarcopenia and NAFLD disease severity [20].

We hypothesised that there would be an inverse relationship between FLI and skeletal muscle density. The aim of this study was to investigate the association between liver fat and muscle fat, using FLI and skeletal muscle density as surrogate markers of fat accumulation in these organs. We also investigated associations between the FLI and parameters of sarcopenia.

## Methods

### Participants

This cross-sectional analysis utilises data from men enrolled in the Geelong Osteoporosis Study (GOS), a population-based prospective cohort study of men and women, set in southeastern Australia. The GOS recruited 1540 men at random from electoral rolls between 2001 and 2006, with 67% response. The inclusion criterion was a listing on the electoral roll as a resident of the Barwon Statistical Division; residency in the region for less than 6 months and/or inability to provide informed consent necessitated exclusion. Most (~ 98%) of the participants were white. Details of recruitment and characteristics of the cohort have been published elsewhere [21]. Follow-up assessments occurred 5, 6, and 15 years later. Data for this analysis were collected at the most recent follow-up, 2016–2020. From a potential 625 men who participated in this phase, 403 (ages 33–96 years) provided a blood sample and underwent anthropometry and other clinical assessments including valid peripheral quantitative computed tomography (pQCT), and were thus included in this analysis.

The study was approved by the Human Research Ethics Committee at Barwon Health. All participants provided written informed consent.

### Muscle Density

Muscle density and cross-sectional area (CSA) were measured using pQCT (XCT 2000, Stratec Medizintechnik, Pforzheim, Germany). Standard transverse scans were performed at 66% of radial (*n* = 349) and tibial (*n* = 347) length, and scans were analysed using BonAlyse software (BonAlyse Oy, Jyvaskyla, Finland). The following density thresholds identified bones, muscle, and fat tissue: fat < 15 mg/mm^3^, muscle 15–180 mg/mm^3^, and bone > 180 mg/mm^3^.

### Other Clinical Measures

Body mass was measured to ± 0.1 kg using electronic scales and height was measured to ± 0.01 m using a wall-mounted Harpenden stadiometer; body mass index (BMI) was calculated as body mass/height^2^ (kg/m^2^). Waist circumference was measured in a horizontal plane with a narrow, non-elastic tape measure.

Handgrip strength (HGS) was measured using a hand-held dynamometer (Vernier, LoggerPro3, USA). The maximum value of repeated measures for each hand was used in analyses and values transformed to Jamar equivalent values as previously described [22]. Gait speed was measured over a distance of 4 m. The Timed Up-&-Go (TUG) test over a distance of 3 m was used as a measure of balance and functional mobility [23]. Falls during the previous 12 months were documented by questionnaire and fallers were identified if one or more falls were reported.

Whole body dual energy x-ray absorptiometry (DXA; Prodigy Pro, Lunar, Madison, WI, USA) was used to determine whole body lean and fat mass. Lean mass of the arms and legs were summed to represent appendicular lean mass (ALM), which was expressed relative to height (ALM/h^2^, kg/m^2^) or BMI (ALM/BMI, m^2^). Body fat was expressed as a percentage of total body tissue (%BF). The android-to-gynoid ratio (AGR) was calculated as the ratio of android fat mass (the central region around the waist) and gynoid fat mass (mainly around the hips and thighs). All clinical measures were performed by trained personnel.

### Biomarkers

Blood samples were collected following an overnight fast and sera were stored at − 80 °C until batch analysis. Serum triglycerides, gamma-glutamyltransferase (GGT), and plasma glucose were analysed using standard laboratory methods. Serum levels of interleukin (IL)-6 and tumour necrosis factor (TNF)-α were assayed using a custom-designed Human High Sensitivity T Cell magnetic bead panel (MPHSTCMAG28SK05; Merk Millipore, Bayswater, VIC, AUS), in accordance with the manufacturer’s instructions.

### Lifestyle

Details of medication use and lifestyle were documented by self-report. Alcohol consumption was estimated using the Cancer Council Victoria food frequency questionnaire [24] and high intakes recognised if the average consumption exceeded 30 g/day [25]. Tobacco smoking referred to current use. Mobility was described as ‘active’ if vigorous or light exercise was performed regularly; otherwise individuals were classified as ‘sedentary’, as previously described [21]. Diabetes was identified by fasting plasma glucose ≥ 7.0 mmol/L, use of an antihyperglycemic agent and/or self-report.

### Fatty Liver Index

We used the Fatty Liver Index (FLI) as an indicator of liver fat accumulation [26]. The FLI was calculated from measures of BMI, waist circumference, triglycerides, and GGT to produce a score between 1 and 100 as follows:

$${\text{FLI}} = {{\left( {e^{{0.{953}*\log e\left( {{\text{triglycerides}}} \right) + 0.{139}*{\text{BMI}} + 0.{718}*\log e\left( {{\text{GGT}}} \right) + 0.0{53}*{\text{waist circumference}} - {15}.{745}}} } \right)} \mathord{\left/ {\vphantom {{\left( {e^{{0.{953}*\log e\left( {{\text{triglycerides}}} \right) + 0.{139}*{\text{BMI}} + 0.{718}*\log e\left( {{\text{GGT}}} \right) + 0.0{53}*{\text{waist circumference}} - {15}.{745}}} } \right)} {\left( {{1} + e^{{0.{953}*\log e\left( {{\text{triglycerides}}} \right) + 0.{139}*{\text{BMI}} + 0.{718}*\log e\left( {{\text{GGT}}} \right) + 0.0{53}*{\text{waist circumference}} - {15}.{745}}} } \right)}}} \right. \kern-\nulldelimiterspace} {\left( {{1} + e^{{0.{953}*\log e\left( {{\text{triglycerides}}} \right) + 0.{139}*{\text{BMI}} + 0.{718}*\log e\left( {{\text{GGT}}} \right) + 0.0{53}*{\text{waist circumference}} - {15}.{745}}} } \right)}}*{1}00$$

The distribution of FLI values were skewed, so the values were categorised into quartiles using the cut-points 0.597, 1.759, and 4.971, which corresponded to 25%, 50%, and 75% of the distribution; quartile (Q) 1 was designated low FLI. A FLI < 30 (negative likelihood ratio = 0.2) rules out and a FLI ≥ 60 (positive likelihood ratio = 4.3) rules in NAFLD.

### Statistical Methods

Aggregate descriptive statistics were used to describe participant characteristics, and differences between FLI quartiles were identified using analysis of variance (ANOVA) where continuous data were normally distributed and Mann–Whitney *U* analysis where continuous variables deviated from the normal distribution. The Chi-square test was used to identify differences between categorical data. Linear regression models (analysis of covariance, ANCOVA) were developed to determine how muscle density at the radial and tibial sites, ALM/h^2^, ALM/BMI and HGS differed across quartiles of FLI. Binary logistic regression models were utilised to detect the likelihood of sarcopenia (based on low ALM/BMI and low HGS) for each quartile of FLI. Potential covariates included age, high alcohol iαntake, smoking, sedentary lifestyle, use of lipid-lowering medication, and serum inflammatory markers (natural log transformed to normalise the data); models for muscle density were also adjusted for muscle CSA and ALM. Final models were tested for effect modification. The association between FLI (quartiles) and muscle density were repeated in two sensitivity analyses that excluded potential effects of diabetes and a high alcohol intake on this relationship. All analyses were performed using Minitab (v16, Minitab, State College, PA, USA).

## Results

### Sample Characteristics

The range of FLI values in each quartile was Q1 (lowest quartile, *n* = 101) 0.04–0.60, Q2 (*n* = 101) 0.61–1.75, Q3 (*n* = 101) 1.76–4.97, and Q4 (*n* = 100) 5.35–84.0.

Participant characteristics are shown in Table 1. Indices of adiposity, including mean BMI, waist circumference, fat mass, %BF, AGR, and median triglyceride and GGT levels increased across increasing FLI quartiles; no interquartile differences were detected in median age. The proportions of participants with diabetes increased across increasing FLI quartiles and this was also the pattern for sedentary lifestyles, slow TUG times, use of a lipid-lowering medication, and a high alcohol intake. No interquartile differences were observed for individuals with a fall in the previous 12 months.

**Table 1:** Participant characteristics for the whole group and according to quartiles of fatty liver index (FLI)

	All *n* = 403	Fatty liver index (FLI)				
		Q1 *n* = 101	Q2 *n* = 101	Q3 *n* = 101	Q4 *n* = 100	*p*
Age (year)	64.7 (54.0–73.5)	62.6 (49.3–74.6)	63.2 (55.2–72.7)	66.1 (55.1–72.8)	67.0 (56.3–73.6)	0.287
BMI (kg/m^2^)	27.8 (± 4.0)	23.6 (± 1.9)	26.6 (± 1.9)	28.4 (± 1.8)	32.5 (± 3.7)	< 0.001
Waist (cm)	100 (± 13)	87 (± 7)	96 (± 5)	103 (± 6)	115 (± 13)	< 0.001
Triglycerides (mmol/L)	1.2 (0.9–1.8)	0.9 (0.7–1.1)	1.1 (0.9–1.5)	1.5 (1.0–2.0)	2.0 (1.5–2.6)	< 0.001
GGT (U/L)	24 (17–35)	16 (13–23)	22 (17–28)	27 (20–39)	35 (25–63)	< 0.001
IL-6 (pg/mL)	1.40 (0.73–2.54)	1.58 (0.73–2.91)	1.27 (0.68–2.41)	1.39 (0.92–2.48)	1.47 (0.69–2.31)	0.514
TNF-α (pg/mL)	4.09 (3.18–5.18	3.75 (2.83–5.00)	4.13 (3.38–5.09)	4.10 (3.13–5.31)	4.22 (3.05–5.36)	0.608
Fat mass (kg)	25.4 (± 9.4)	15.3 (± 4.9)	23.3 (± 4.9)	27.0 (± 5.1)	36.0 (± 8.0)	< 0.001
%BF (%)	30.1 (± 7.5)	21.8 (± 6.0)	29.2 (± 5.1)	32.3 (± 4.6)	37.1 (± 4.5)	< 0.001
AGR	0.70 (± 0.17)	0.57 (± 0.17)	0.70 (± 0.13)	0.75 (± 0.15)	0.79 (± 0.14)	< 0.001
Diabetes*	44 (11.5%)	5 (5.3%)	3 (3.2%)	19 (20.0%)	17 (17.5%)	< 0.001
Use of lipid-lowering medication	113 (28.0%)	14 (13.9%)	28 (27.7%)	30 (29.7%)	41 (41.0%)	< 0.001
Smokers	28 (6.9%)	9 (8.9%)	8 (7.9%)	9 (8.9%)	2 (2.0%)	0.162
Alcohol > 30 g/day	81 (20.1%)	9 (8.9%)	23 (22.8%)	23 (22.8%)	26 (26.0%)	0.012
Sedentary behaviour	96 (23.8%)	15 (14.9%)	17 (16.8%)	28 (27.7%)	36 (36.0%)	0.001
Gait speed (m/s)	0.96 (± 0.21)	1.00 (± 0.23)	0.99 (± 0.21)	0.94 (± 0.20)	0.90 (± 0.20)	0.003
Gait speed* < 0.8 m/s	48 (12.0%)	11 (10.9%)	8 (8.1%)	13 (13.0%)	16 (16.0%)	0.367
TUG (s)	8.4 (7.5–9.8)	8.0 (7.2–9.1)	8.1 (7.3–9.3)	8.4 (7.8–10.0)	9.2 (8.0–10.8)	< 0.001
TUG > 10 s	62 (15.4%)	12 (11.9%)	7 (6.9%)	16 (15.8%)	27 (27.0%)	0.001
Faller	54 (13.4%)	10 (9.9%)	11 (10.9%)	16 (15.8%)	17 (17.0%)	0.355

Data are shown as mean (± standard deviation), median (interquartile range) or *n* (%)

*BMI* body mass index; *GGT* gamma-glutamyltransferase; *AGR* android fat mass to gynoid fat mass ratio; *%BF* body fat percentage

*Missing data: diabetes *n* = 21; gait speed *n* = 3

### FLI and Components of Sarcopenia

There was a pattern of increasing ALM/h^2^, and decreasing ALM/BMI, across FLI quartiles (Table 2). The relationships between increasing FLI quartiles with increasing ALM/h^2^ and decreasing ALM/BMI persisted after adjusting for age and sedentary lifestyle (Table 3). No interquartile differences in HGS were observed.

**Table 2:** Skeletal muscle characteristics for the whole group and according to quartiles of fatty liver index (FLI)

	All *n* = 403	Fatty liver index (FLI)				
		Q1 *n* = 101	Q2 *n* = 101	Q3 *n* = 101	Q4 *n* = 100	*p*
Radius: muscle density (mg/cm^3^)	76.21 (± 2.77)	76.96 (± 2.39)	76.58 (± 2.36)	76.42 (± 2.19)	74.92 (± 3.50)	< 0.001
Tibia: muscle density (mg/cm^3^)	71.51 (± 4.59)	73.05 (± 4.13)	71.85 (± 4.33)	71.06 (± 4.37)	69.85 (± 5.02)	< 0.001
Radius: muscle CSA (cm^2^)	41.53 (± 6.45)	39.13 (± 6.40)	40.71 (± 5.98)	41.40 (± 5.48)	44.79 (± 6.58)	< 0.001
Tibia: muscle CSA (cm^2^)	71.24 (± 10.51)	67.84 (± 10.31)	70.84 (± 9.91)	71.32 (± 9.84)	75.41 (± 10.82)	< 0.001
ALM (kg)	26.0 (± 3.8)	24.5 (± 3.5)	26.0 (± 3.8)	25.8 (± 3.2)	27.5 (± 4.0)	< 0.001
ALM/h^2^ (kg/m^2^)	8.49 (± 0.94)	8.04 (± 0.85)	8.41 (± 0.92)	8.54 (± 0.79)	8.99 (± 0.95)	< 0.001
Low ALM/h^2^	16 (4.0%)	9 (8.9%)	3 (2.0%)	3 (2.0%)	2 (2.0%)	-
ALM/BMI (m^2^)	0.95 (± 0.15)	1.04 (± 0.14)	0.98 (± 0.13)	0.91 (± 0.11)	0.85 (± 0.13)	< 0.001
Low ALM/BMI	82 (20.4%)	6 (5.9%)	10 (9.9%)	22 (21.8%)	44 (44.0%)	< 0.001
HGS (kg)	40.1 (± 7.2)	40.5 (± 7.5)	41.2 (± 6.9)	39.3 (± 6.8)	39.3 (± 7.3)	0.179
Low HGS*	111 (27.6%)	27 (27.0%)	20 (19.8%)	31 (30.7%)	33 (33.0%)	0.169
Low HGS and low ALM/h^2^*	8 (2.0%)	5 (5.0%)	2 (2.0%)	0 (0%)	1 (1.0%)	-
Low HGS and low ALM/BMI*	40 (10.0%)	4 (4.0%)	6 (5.9%)	9 (8.9%)	21 (21.0%)	< 0.001

Low values: HGS < 35.5 kg; ALM/h^2^ (< 6.94 kg/m^2^), ALM/BMI (< 0.827 m^2^)

*CSA* cross-sectional area; *ALM* appendicular lean mass; *BMI* body mass index; *HGS* handgrip strength

*Missing data: radius muscle density and CSA *n* = 54; tibia muscle density and CSA *n* = 56; HGS *n* = 1

**Table 3:** Linear regression models showing the relationship between fatty liver index (FLI) quartiles (Q1 the lowest, and reference), appendicular lean mass, handgrip strength, and muscle density at the radial and tibial sites.

	FLI	Beta coefficient	SE	*p*
Radius: muscle density (mg/cm^3^)	Q1	Reference		
	Q2	− 0.2589	0.3457	0.454
	Q3	− 0.2349	0.3429	0.494
	Q4	− 1.6623	0.3731	0.000
Tibia: muscle density (mg/cm^3^)	Q1	Reference		
	Q2	− 0.3969	0.5442	0.466
	Q3	− 1.0329	0.5558	0.064
	Q4	− 1.6463	0.6067	0.007
ALM/h^2^ (kg/m^2^)	Q1	Reference		
	Q2	0.4356	0.1106	0.000
	Q3	0.6199	0.1111	0.000
	Q4	1.1094	0.1125	0.000
ALM/BMI (m^2^)	Q1	Reference		
	Q2	− 0.05140	0.01530	0.000
	Q3	− 0.11038	0.01538	0.000
	Q4	− 0.16181	0.01557	0.000
Handgrip strength (kg)	Q1	Reference		
	Q2	1.4482	0.8397	0.085
	Q3	− 0.0707	0.8436	0.933
	Q4	0.1813	0.8538	0.832

Muscle density models were adjusted for muscle CSA, ALM, age and sedentary lifestyle. Other models adjusted for age and sedentary lifestyle

*ALM* appendicular lean mass; *BMI* body mass index

Eight participants (2.0%) had both low HGS and low ALM/h^2^, fulfilling criteria for sarcopenia (Table 2). The numbers of participants in each FLI quartile for sarcopenia using these criteria were too small for meaningful analyses. However, 40 participants (10.0%) had both low HGS and low ALM/BMI. Using these criteria for sarcopenia revealed that participants in the highest FLI quartile (Q4) were sixfold more likely to have sarcopenia than those in the lowest quartile (Q1) (*p* = 0.004). No other interquartile differences were observed. With Q1 as reference, and models adjusted for age and lifestyle, the ORs (95%CIs) for each quartile were: Q2 1.50 (0.37, 6.00); Q3 1.91 (0.51, 7.09); and Q4 6.01 (1.77, 20.4).

### FLI and Muscle Density

**Fig. 1:**
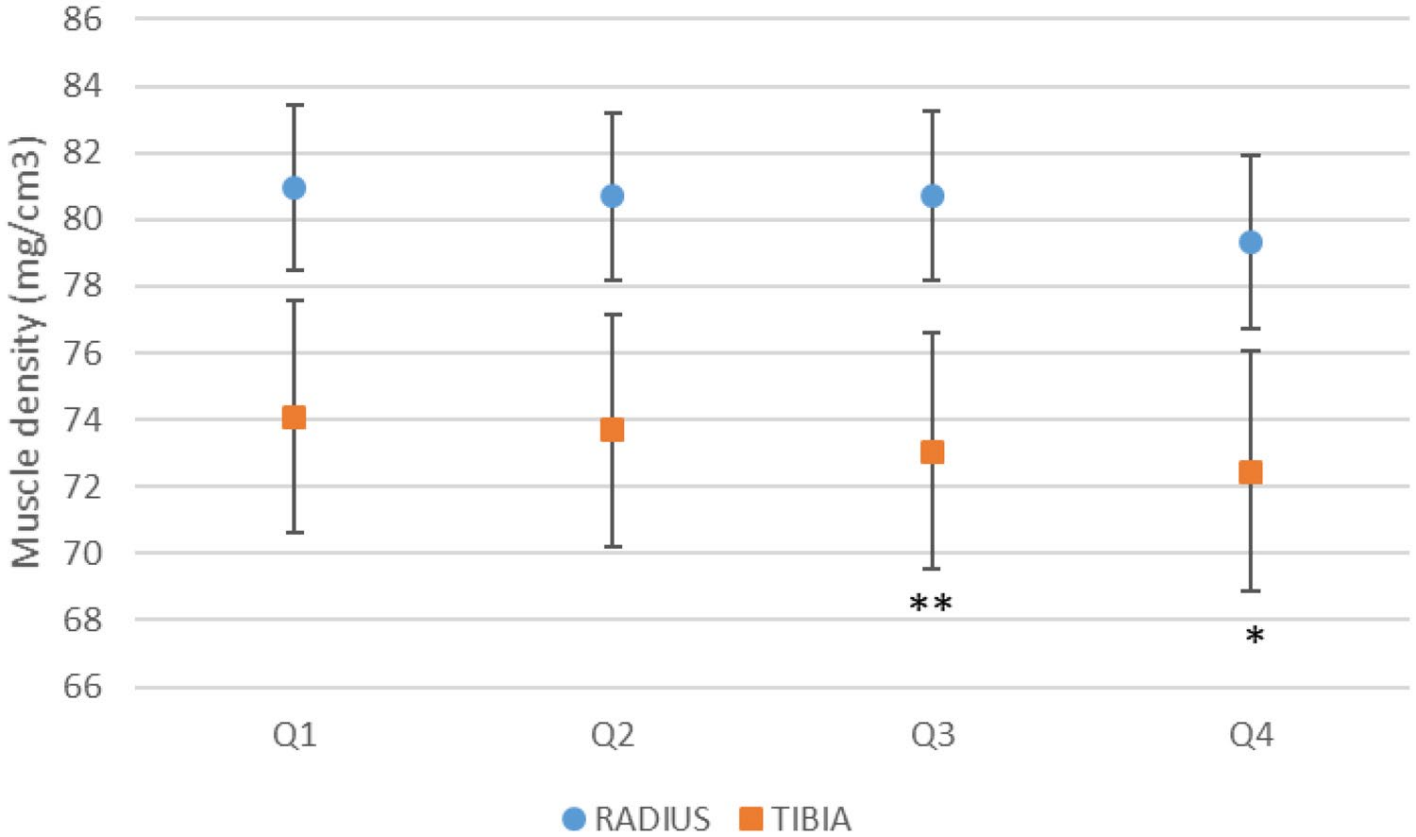
Mean muscle density for each fatty liver index (FLI) quartile (Q); Q1 0.04–0.60, Q2 0.61–1.75, Q3 1.76–4.97, Q4 5.35–84.0. *Fatty Liver Index Q1 (reference) v Q4 *p* ≤ 0.007; and **Q1 (reference) v Q3, *p* = 0.06. The bars represent ± standard error

There was a pattern of decreasing muscle density at the radial and tibial sites across increasing quartiles of FLI (Table 2). This pattern persisted after adjusting for age, sedentary lifestyle, ALM, and muscle CSA (Fig. 1). The associations were not explained by adjusting for use of a lipid-lowering medication, smoking or markers of inflammation. Compared with FLI QI, mean radial muscle density in Q4 was 2.1% lower (*p* < 0.001). Similarly, mean tibial muscle density was 2.2% lower in the highest compared to the lowest quartile (*p* = 0.006) and there was a trend for Q3 to be 0.5% lower than Q1 (*p* = 0.06).

### Sensitivity Analyses

In the first sensitivity analysis, 44 participants with diabetes were excluded. Amongst the remaining 359 participants, adjusted mean muscle density at the radius was lower for the highest FLI quintile compared with lowest (*p* < 0.001). Adjusted mean (95% CI) for each FLI quartile, Q1 v Q2 v Q3 v Q4: 80.46 (77.83, 83.08) v 80.16 (77.53, 82.80) v 80.46 (77.77, 83.15) v 78.81 (76.07, 81.55) mg/cm^3^. For the tibia, mean muscle density was lower for Q4 than Q1 (*p* = 0.027). The mean (95% CI) values for each quartile of FLI: 75.01 (71.54, 78.48) v 74.84 (71.33, 78.35) v 74.30 (70.71, 77.89) v 73.63 (69.99, 77.26) mg/cm^3^.

In the second sensitivity analysis, 81 participants with high alcohol intake were excluded. Amongst the remaining 403 participants, adjusted mean muscle density at the radius was lower for the highest FLI quintile compared with lowest (*p* < 0.001). Adjusted mean (95% CI) for each FLI quartile, Q1 v Q2 v Q3 v Q4: 80.48 (77.69, 83.26) v 80.29 (77.47, 83.10) v 80.27 (77.45, 83.10) v 78.54 (75.64, 81.44) mg/cm^3^. For the tibia, in comparison to Q1, mean muscle density was lower for Q3 (*p* = 0.021) and lower for Q4 (*p* = 0.001). The mean (95% CI) values for each quartile of FLI: 74.94 (71.02, 78.85) v 74.52 (70.53, 78.51) v 73.53 (69.54, 77.52) v 72.65 (68.58, 76.72) mg/cm^3^.

### NAFLD and Muscle Density

Amongst the 322 participants without high alcohol intake, four had FLI ≥ 60 and met criteria for NAFLD, and five had FLI between 30 and 60. Median differences in radial muscle density for those with (*n* = 4) and without (*n* = 273) NAFLD were 75.25 (70.81, 78.28) v 76.61 (74.99, 78.16) mg/cm^3^, *p* = 0.477; values of tibial muscle density for those with (*n* = 3) and without (*n* = 279) NAFLD were 73.83 (67.87, 73.83) v 72.49 (69.30, 74.72) mg/cm^3^, *p* = 0.677. Numbers were too small for multivariable analyses.

## Discussion

We report that men in the highest quartile of FLI had lower muscle density than those in the lowest quartile. Our results suggest that individuals likely to have more fat in the liver also have more fat in their muscle, at least at the radial and tibial regions that we measured. We also found that men in the highest quartile of FLI were more likely to have sarcopenia than those in the lowest quartile.

There is an emerging body of evidence to support links between skeletal muscle composition and chronic liver disease [20, 27]. Using different methodologies, several studies have reported low muscle volume in association with liver steatosis [12–17].

Both liver and muscle store carbohydrate as glycogen, and when storage capacities are overwhelmed, these organs convert excess carbohydrate into fat. Risk factors for fat accumulation in body organs include obesogenic environments, unhealthy lifestyles, and a genetic predisposition [28]. Whilst not limited to individuals with obesity, liver steatosis is more prevalent in those with metabolic aberrations, such as excessive visceral (central) fat accumulation and insulin resistance [2]. Combined low muscle volume and myosteatosis is more prevalent in NAFLD than in other chronic liver diseases [11]. NAFLD can progress to non-alcoholic steatohepatitis (NASH), which increases the risk for cirrhosis and hepatic carcinoma. Hepatic steatosis is interconnected with the metabolic syndrome and associated cardiometabolic diseases [2]; myosteatosis is also associated with chronic endocrine and non-endocrine disease [29], poor cognition [30], and poor mobility [31]. To date there has been little research to unravel potential pathophysiological links between hepatic steatosis and myosteatosis [18, 19], but underlying pathways are likely to involve lipotoxicity, insulin resistance, activated pathways for inflammation and oxidative stress, and mitochondrial dysfunction [28, 31–34].

In a study of 309 men and women (mean age 53 ± 14 years) from the Boramae NAFLD Registry in South Korea, individuals with sarcopenia, defined as low appendicular skeletal muscle index (measured by bioelectrical impedance analysis, BIA, and expressed as a percentage of body mass), were more likely to have NASH and significant fibrosis, independent of obesity, insulin resistance, and inflammation [20]. The authors concluded that sarcopenia was inversely associated with histological severity in NAFLD. A study using data from the National Health and Nutrition Examination Survey III (NHANES III) in the USA involving 2551 participants aged 60–75 years identified NAFLD by ultrasound, and sarcopenia by low appendicular skeletal muscle index (by BIA; indexed to height or body mass) and function [35]. They reported that severe hepatic steatosis was inversely associated with sarcopenia using height-adjusted skeletal muscle index (kg/m^2^), but positively associated with sarcopenia using weight-adjusted skeletal muscle index (kg/kg).

A recent report from the Rotterdam Study described the inter-relationships between DXA-derived lean mass, body fat mass and distribution, and ultrasound-derived liver steatosis and NAFLD in 4609 elderly men and women from the Netherlands [36]. They reported that body fat mass, particularly android fat mass, was a better predictor than low lean mass for NAFLD. A negative association between ALM/h^2^ and NAFLD was independent of metabolic confounders and fat distribution in normal-weight women, but not men. No association was observed between sarcopenia and NAFLD. In our cross-sectional analysis of data for men, we report that indices of adiposity, central fat accumulation and ALM/h^2^ increased, and ALM/BMI decreased, across increasing quartiles of FLI. No association was detected between quartiles of FLI and handgrip strength or gait speed.

The absence of an internationally accepted operational definition for sarcopenia has led to inconsistencies in the way sarcopenia is identified [37]. In particular, diagnostic methods for low muscle quantity include total lean mass or ALM adjusted for height (kg/m^2^), BMI (m^2^), or body mass (%), and not all definitions include parameters of muscle function. Where muscle dysfunction attributed to low muscle volume occurs in the face of obesity, the combination is referred to as sarcopenic obesity [38, 39]; age-related muscle deterioration accompanied by increases in body fat mass has been identified [40]. Peng et al. [35, 41] noted that BMI confounds the relationship between sarcopenia and NAFLD in studies using different definitions for sarcopenia. We also know that BMI is limited as an estimate of body fatness, and it varies with age, sex, and ethnicity [42, 43].

In this study, we evaluated skeletal muscle density using pQCT. This imaging technology evolved from the established computed tomography (CT) to quantify bone parameters at peripheral sites and, more recently, for quantifying muscle mass and fat distribution. In muscles, both modalities use x-ray beam attenuation to distinguish fat from fat-free mass. CT-derived muscle density at the mid-thigh has been negatively correlated with lipid content from percutaneous biopsy specimens [3]. As with CT, pQCT determines the CSA of soft tissue and estimates muscle density. The main advantage of using pQCT instead of CT is the extremely low effective radiation dose and shorter scan time [44], making it suitable for epidemiological studies.

We acknowledge several strengths and weaknesses in our study. Particular strengths are that participants were representative of the general adult population and not selected on the basis of disease. We did not have a direct measure of liver fat, but used the FLI as a surrogate. In a study showing that the FLI discriminated between patients with and without liver steatosis, a poor relationship was reported between FLI and liver fat measured by proton magnetic resonance spectroscopy [45]. On the other hand, a recent community-based study indicated a correlation (*r* = 0.56–0.59) between liver fat estimated by ultrasound and the FLI [46]. We used DXA-derived ALM as a surrogate for skeletal muscle mass which has been shown to indicate a correlation (*r* = 0.91 for the leg) with skeletal muscle mass quantified by magnetic resonance imaging [47]. The use of the FLI to stratify participants is a potential limitation in analyses involving indices of adiposity, for example ALM/BMI, %BF, waist circumference, as they are part of the FLI. The FLI stratification may have also limited our ability to identify associations between inflammatory markers, hepatic steatosis, and myosteatosis. Furthermore, our results are likely to depend on the definitions and cut-points selected a priori for identifying low values for muscle parameters and sarcopenia for the secondary analyses regarding associations between FLI and muscle mass, strength, and performance. Although we have accounted for indices of adiposity in the FLI, there is likely to be residual confounding in our statistical models. Further, we used cross-sectional data, which cannot infer causality. Our data were pertinent for men residing in southeastern Australia, and may not be generalisable to other populations of men, nor to women.

Given these constraints, we conclude that fat accumulation in the liver co-exists with fat infiltration into skeletal muscle. The presence of abnormal liver function tests or NAFLD diagnosis should prompt assessment for sarcopenia and frailty (using muscle performance tests) as a clinical consequence of myosteatosis. On the other hand, identification of poor muscle function associated with low muscle density may indicate the presence of unrecognised hepatosteatosis. Storage of ectopic fat in liver and skeletal muscle, leading to fatty liver disease and compromised skeletal muscle, places individuals in a web of metabolic disturbances in combination with muscle deficits that are likely to increase the risk for poor physical function.

## Data Availability

Data will be made available upon reasonable request.
